# Smartphone-readable RPA-LFA for the high-sensitivity detection of Leishmania kDNA using nanophosphor reporters

**DOI:** 10.1371/journal.pntd.0011436

**Published:** 2023-07-03

**Authors:** Adheesha N. Danthanarayana, Suman Nandy, Katerina Kourentzi, Binh Vu, Thomas R. Shelite, Bruno L. Travi, Jakoah Brgoch, Richard C. Willson

**Affiliations:** 1 Department of Chemistry, University of Houston, Houston, Texas, United States of America; 2 William A. Brookshire Department of Chemical and Biomolecular Engineering, University of Houston, Houston, Texas, United States of America; 3 Department of Biosafety, University of Texas Medical Branch, Galveston, Texas, United States of America; 4 Department of Internal Medicine, University of Texas Medical Branch, Galveston, Texas, United States of America; 5 Department of Biology and Biochemistry, University of Houston, Houston, Texas, United States of America; Institute of Tropical Medicine, BELGIUM

## Abstract

Early diagnosis of infectious diseases improves outcomes by enabling earlier delivery of effective treatment, and helps prevent further transmission by undiagnosed persons. We demonstrated a proof-of-concept assay combining isothermal amplification and lateral flow assay (LFA) for early diagnosis of cutaneous leishmaniasis, a vector-borne infectious disease that affects *ca*. 700,000 to 1.2 million people annually. Conventional molecular diagnostic techniques based on polymerase chain reaction (PCR) require complex apparatus for temperature cycling. Recombinase polymerase amplification (RPA) is an isothermal DNA amplification method that has shown promise for use in low-resource settings. Combined with lateral flow assay as the readout, RPA-LFA can be used as a point-of-care diagnostic tool with high sensitivity and specificity, but reagent costs can be problematic. In this work, we developed a highly-sensitive smartphone-based RPA-LFA for the detection of *Leishmania panamensis* DNA using blue-emitting [(Sr_0.625_Ba_0.375_)_1.96_Eu_0.01_Dy_0.03_]MgSi_2_O_7_ (SBMSO) persistent luminescent nanophosphors as LFA reporters. The greater detectability of nanophosphors allows the use of a reduced volume of RPA reagents, potentially reducing the cost of RPA-LFA. The limit of detection (LOD) of RPA with gold nanoparticle-based LFA readout is estimated at 1 parasite per reaction, but LOD can be 100-fold better, 0.01 parasites per reaction, for LFA based on SBMSO. This approach may be useful for sensitive and cost-effective point-of-care diagnosis and contribute to improved clinical and economic outcomes, especially in resource-limited settings.

## Author summary

The early diagnosis of cutaneous leishmaniasis decreases morbidity and avoids long-term complications. We have developed a smartphone-based test for cutaneous leishmaniasis combining recombinase polymerase amplification with LFA using [(Sr_0.625_Ba_0.375_)_1.96_Eu_0.01_Dy_0.03_]MgSi_2_O_7_ nanophosphors as reporters. The nanophosphor-based LFA shows 50 to 100-fold greater sensitivity than gold nanoparticle-based LFAs in detecting *Leishmania* RPA products. Furthermore, this method can achieve high sensitivity using a minimal volume (2 μL) of RPA reaction, significantly reducing reagent usage and cost. This approach is promising for the rapid, sensitive, and cost-effective point-of-care diagnosis of cutaneous leishmaniasis, especially in resource-limited settings.

## Introduction

Cutaneous leishmaniasis is a vector-borne disease caused by protozoan parasites of the genus *Leishmania* and is transmitted by the bite of an infected female sand fly. Cutaneous leishmaniasis generally presents as ulcerated skin lesions, often leaving lifelong scars and associated stigma, and may result in severe disability. Sand flies transmitting cutaneous leishmaniasis are found throughout Central and South America, in southern Europe, and in some parts of Asia and Africa, impacting an estimated 700,000 to 1.2 million or more people annually.[1,2] Cutaneous leishmaniasis is a treatable disease, and early diagnosis could decrease morbidity by preventing the development of large dermal lesions. Additionally, it could limit outbreaks caused by transmission *via* undiagnosed people.

Polymerase chain reaction (PCR)-based diagnosis of cutaneous leishmaniasis has high sensitivity and specificity, enabling identification to the species level and allowing specific treatment. Cutaneous leishmaniasis has been detected by kinetoplast DNA (kDNA)-targeted PCR with 98.7% sensitivity.[3] However, PCR tests require thermal cyclers for highly accurate temperature control and must be conducted by trained personnel, limiting access for people in endemic regions with limited infrastructure.[4] Cutaneous leishmaniasis can also be diagnosed by conventional direct microscopy or culture of skin specimens, but these methods are relatively insensitive and time consuming, and require trained personnel.[1,5–7] Therefore, there is a huge need for point-of-care (POC) tests for early diagnosis of the disease that meet the needs of target population and the requirements for implementation in resource-limited settings where most cases occur [8].

A promising alternative to PCR is recombinase polymerase amplification (RPA), an isothermal nucleic acid amplification technique requiring minimal equipment and sample preparation. End-point RPA is most commonly read by lateral flow assay (LFA) in POC settings [9]. For example, RPA-LFA has recently been used to develop rapid diagnostic tests for SARS-CoV-2 [10,11]. In addition, Rivas *et al*. developed an RPA-LFA workflow using a triple-line gold nanoparticle-based LFA for the detection of *Leishmania infantum* DNA in dog blood samples with a limit of detection (LOD) of 0.038 spiked *Leishmania* parasites per DNA amplification reaction (1 parasite/100 μL of DNA sample) [12]. We previously developed an RPA-LFA workflow for detecting cutaneous *Leishmania Viannia spp*. infection using a gold nanoparticle-based LFA with an analytical sensitivity equivalent to 0.1 parasites per reaction [5]. Furthermore, we recently tested the diagnostic performance of a gold nanoparticle-based RPA-LFA for cutaneous leishmaniasis using 118 patient samples in an endemic setting in Colombia and found 87% sensitivity and 86% specificity in the reference lab scenario, and 75% sensitivity and 89% specificity in the field scenario [13].

**Fig 1:**
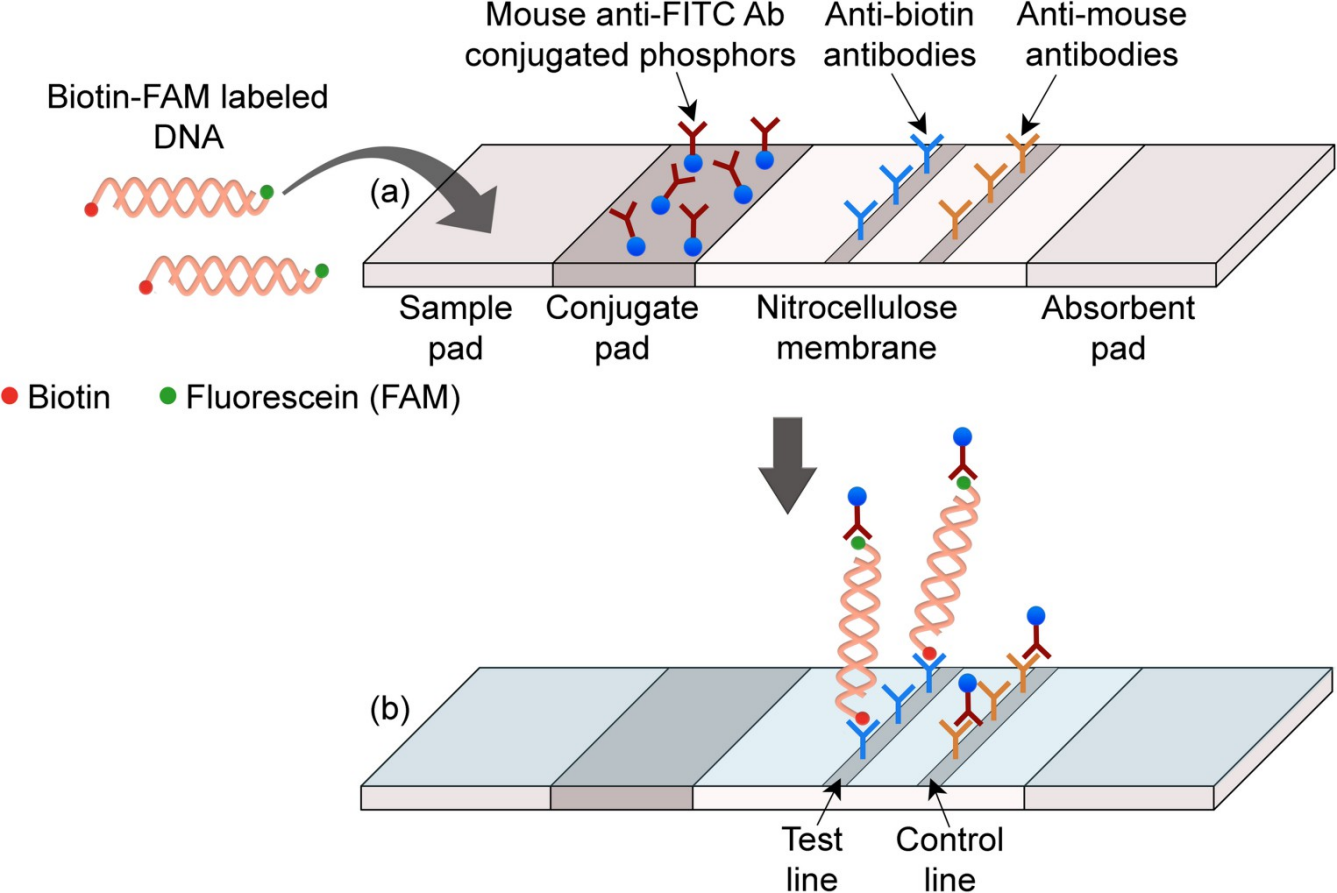
LFA architecture used to detect RPA products. (A) DNA amplicons labeled with biotin and fluorescein (FAM) during the RPA reaction first bind to reporter particles conjugated with monoclonal mouse anti-FITC antibodies in the conjugate pad. (b) The complexes then travel along the membrane and are captured by anti-biotin antibodies immobilized at the test line. Anti-mouse antibodies capture the excess reporters at the control line. Positive samples show both the test and the control lines; negative samples show only a control line.

In this study, we demonstrate a novel approach to a highly sensitive and economical RPA-LFA workflow by replacing gold nanoparticle LFA reporters with persistent luminescent nanophosphors. [(Sr_0.625_Ba_0.375_)_1.96_Eu_0.01_Dy_0.03_]MgSi_2_O_7_ (SBMSO) is a blue-emitting solid-state inorganic compound substituted with rare-earth ions Eu^2+^ and Dy^3+^. When excited with UV/blue light, it emits long-lasting luminescence (>9 min) even after the termination of excitation. The long luminescent lifetime allows smartphone-based time-resolved measurements with a 100 ms time delay between excitation and capture of emitted luminescence using a standard smartphone camera, leading to reduced background from excitation and/or autofluorescence, increasing sensitivity, and avoiding the need for delicate, expensive spectral filters [14]. Compared to conventional LFA reporters such as gold nanoparticles, organic fluorophores, and quantum dots, persistent luminescent nanophosphors not only allow for enhanced detection sensitivity, but they also are resistant to photobleaching and can use very inexpensive reading hardware [15–17]. In this study, we extend the use of SBMSO nanophosphors to the detection of nucleic acids and demonstrate their superior sensitivity compared to commonly-used gold nanoparticles in the detection of *Leishmania panamensis* DNA by RPA-LFA using the LFA architecture shown in Fig 1. We also combined RPA-LFA with smartphone-based imaging towards the development of a user-friendly and straightforward POC test for cutaneous leishmaniasis.

## Materials and methods

### Recombinase polymerase amplification of *Leishmania Viannia sp.*

All evaluations were performed using archived purified DNA of *Leishmania* (*Viannia*) *panamensis* (HOM/COL/84/1099), 100 μL of 6x10^4^ parasites/μL (isolated from a patient in Colombia) and delivered to UTMB by Dr. Nancy Saravia (CIDEIM *Leishmania* repository, Cali, Colombia). *L*. *panamensis* promastigotes were cultured at 28°C in M199 (Gibco, Grand Island, NY) media supplemented with 0.12 mM adenine, 0.0005% hemin, and 20% fetal bovine serum (FBS), and DNA was extracted using the DNeasy Blood & Tissue kit (Qiagen) according to the manufacturer’s protocol [18]. The design of RPA primers amplifying the *Leishmania* kDNA minicircle conserved sequence has been described previously [5]. The biotin-labeled forward primer (5′-biotin-GATGAAAATGTACTCCCCGACATGCCTCTG-3′) and FAM-labeled reverse primer (5′-FAM-CTAATTGTGCACGGGGAGGCCAAAAATAGCGA-3′) (Integrated DNA Technologies) sequence specificities were confirmed *in silico* by BLAST searches of *Leishmania Viannia* subgenus. End-point RPA reactions (at 40°C, 40 min) were carried out in a CFX OPUS Real-Time qPCR System (Bio-Rad) using TwistAmp Basic reagents (TwistDx) and labeled forward and reverse primers. It is important to note that the TwistAmp Basic kit was used instead of the TwistAmp nfo kit, (the nfo probe reduces non-specific amplification [19]) since the nfo kit was not available for purchase when these experiments were conducted. Betaine was added to the RPA reaction mixture to minimize false positives and to reduce non-specific amplification, as previously described [20,21].

Briefly, each lyophilized RPA enzyme pellet was rehydrated with 29.5 μL of primer-free rehydration buffer and mixed with 2.5 μL of each primer (10 μM). Purified *Leishmania* parasite DNA (6 X 10^4^ parasites/μL) was diluted to 120 parasites/μL and DNA was quantified by Nanodrop (2 ng/μL). An amount of 10 ng (5 μL) of *Leishmania* DNA (equivalent to ~600 parasites) was used as the template for the test sample, and 10 ng of Vero cell DNA (purified from green monkey kidney cells) was used as the template for the negative control. The test samples (final concentration: 0.2 ng DNA/μL) and negative controls (final concentration: 0.2 ng DNA/μL) were prepared in nuclease-free water containing 3 M betaine (Sigma-Aldrich) to reduce non-specific amplification, and 3 M betaine was added to bring the total volume up to 47.5 μL. Lastly, 2.5 μL of 280 mM magnesium acetate was added to each tube and mixed in to start the reaction. The tubes were incubated at 40°C for 40 min, and then each 50 μL RPA reaction was divided into two 25 μL fractions. One fraction was purified with a silica membrane-based QIAquick PCR Purification Kit (Qiagen) according to the manufacturer’s recommendation. The other fraction was left unpurified to compare LFA performance with the purified product and to determine if purification can be skipped to simplify the procedure and make the technique more convenient as a POC test. The DNA amplicons were stored at -20°C until use. 1.5% agarose gel electrophoresis in 1X TAE (Tris/acetic acid/EDTA) buffer was performed to confirm the presence of DNA amplicons. 10 μL of the amplified product was combined with 2 μL of 6X Gel Loading Dye, Purple (New England BioLabs Inc.) and 2 μL of prediluted (1,000-fold) SYBR Green I (Thermo Fisher Scientific), incubated briefly, and added to each gel well. The gel was run for ~1 h at 100 V. A Quick-Load 100 base pair DNA ladder (New England BioLabs Inc.) was used as standard.

### Quantification of parasite DNA amplicons

The double-stranded DNA (dsDNA) amplicons were quantified using the QuantiFluor dsDNA system (Promega). A Lambda dsDNA standard curve (0.05–200 ng/well) was prepared in 1X TE buffer. QuantiFluor dsDNA dye was diluted 1:400 in 1X TE buffer and 200 μL of QuantiFluor dsDNA dye was added to each well of a multiwell plate (Corning 96-well flat black) intended for unknown, blank, or standard samples. A volume of 10 μL of prepared dsDNA standard was added to each respective standard well, while 10 μL of 1X TE buffer was added to each blank well, and 4 μL of 5X diluted unknown samples were added to each unknown sample well. The plate was then covered with foil and mixed on a plate shaker. After 5 min, the fluorescence was measured at 504 nm_Ex_/531 nm_Em_ using a plate reader (Infinite Pro M200, Tecan) and the dsDNA concentration of the unknown samples was estimated from the Lambda DNA standard curve.

### Preparation of LFA strips

LFA strips (Fig 1) consisting of Standard 14 sample pad, FF80HP nitrocellulose membrane and CF-5 absorbent pad (all from Cytiva) were assembled on an adhesive backing card (MIBA-020; DCN Diagnostics). Test line (TL) and control line (CL) were striped on the nitrocellulose membrane with 1 mg/mL polyclonal goat anti-biotin antibodies (ab 6643; Abcam) and 1 mg/mL polyclonal goat anti-mouse antibodies (ABGAM-0500; Arista Biologicals, Inc.), respectively, using a BioDot dispenser (XYZ30600124) at a rate of 1 μL/cm. The striped membrane was dried at 37°C for 30 min in an incubator (Robbins Scientific Micro Hybridization Incubator 2000) and then cut into 3 mm wide strips using a ZQ2000 Guillotine Cutter (Kinbio). LFA strips were stored dried (in plastic tubes with desiccant bags) until use.

### Synthesis and functionalization of SBMSO reporters

Polycrystalline powder of [(Sr_0.625_Ba_0.375_)_1.96_Eu_0.01_Dy_0.03_]MgSi_2_O_7_ (SBMSO) was synthesized by high-temperature solid-state synthesis, as described in our previous work [16]. Briefly, a stoichiometric hand-ground mixture of SrCO_3_, BaCO_3_, MgO, SiO_2_, Eu_2_O_3_, and Dy_2_O_3_ with 5 wt.% H_3_BO_3_ was pressed into a pellet and heated at 1,150°C for 6 h in a reducing atmosphere of 5% H_2_/95% N_2_ with heating and cooling rates of 3°C/min. The powder was reground and sintered again at 1,000°C for 4 h with the same reducing atmosphere and ramp rates as the initial heating. Particle size was reduced by ball milling, and particles of ~250 nm were isolated by differential centrifugal sedimentation in anhydrous ethanol.

Particles were then silica-encapsulated using a modified Stöber process as described previously [16]. Briefly, a solution of 221.6 μL of anhydrous ethanol, 246.7 μL of DI water, and 6.7 μL of tetraethyl orthosilicate (TEOS) was added to 1 mL of SBMSO (2 mg/mL). Next, the mixture was sonicated in a bath sonicator (Fisher Scientific FS30) for 5 min, and 25 μL of 30% aqueous ammonium hydroxide was added to the suspension, followed by sonication for another 30 min. The tube with nanophosphors was then placed on a rotator at 20 rpm at room temperature for 7.5 h. Finally, the particles were washed three times with 1 mL of anhydrous ethanol.

Particles were conjugated with antibodies by reductive amination chemistry [16]. A volume of 1 mL of silica-encapsulated nanophosphors dispersed in ethanol (2 mg/mL) in a 2 mL microcentrifuge tube was centrifuged for 3 min at 3000 rcf. The top 216 μL of the ethanol was removed, and 10 μL of a mixture of 155 μL of TEOS, 5 μL of triethoxysilylbutyraldehyde (TESBA), and 1,393 μL of anhydrous ethanol was added to the nanophosphor solution, followed by 189 μL of DI water and 16.7 μL of aqueous ammonium hydroxide. The mixture was sonicated for 10 min in a bath sonicator and then placed on a rotator at 20 rpm at room temperature for 12 h. The particles were then washed with 1 mL of anhydrous ethanol at least three times.

The nanophosphors were washed with DI water and phosphate-buffered saline, pH 8 (PBS), then re-suspended in 700 μL of PBS, pH 8 and sonicated for 5 min. 50 μL of 1 mg/mL monoclonal mouse anti-FITC antibodies (ab112511; Abcam) was added and the solution was vortexed. A volume of 250 μL of 1 M NaBH_3_CN in PBS, pH 8 was added, and the solution was sonicated for 5 min and then placed on a rotator at 20 rpm for 2 h at room temperature.

The nanophosphors were then washed with PBS, pH 7.4, and re-suspended in 200 μL of PBS. For passivation, 750 μL of 80 mg/mL bovine serum albumin (BSA) in PBS, pH 7.4, and 50 μL of 1 M NaBH_3_CN were added to the nanophosphor suspension. After 5 min sonication, nanophosphors were placed on a room temperature rotator for 3 h at 20 rpm followed by washing three times with PBS, pH 7.4. The nanophosphors were subsequently resuspended in 100 μL of borate storage buffer (10 mM sodium borate, 150 mM NaCl, 0.1% BSA, 0.04% polyvinylpyrrolidone (PVP-40; 40,000 avg. mol. wt.), 0.025% Tween 20, pH 8.5) and stored at 4°C.

### Detection of parasite DNA amplicons on lateral flow assay

*Gold LFA*. Serial dilutions of both purified and unpurified DNA amplicons (0–20 ng/mL) were run on commercial gold-nanoparticle LFA strips designed to detect biotin- and FITC/FAM-labeled amplicons (Milenia HybriDetect 1; TwistDx). A volume of 2 μL of DNA sample was mixed with 48 μL of the manufacturer-supplied running buffer. The mixture was added to the sample pad, and the colored lines were observed by eye after 5 min.

*Nanophosphor LFA*. Serial dilutions of both purified and unpurified DNA amplicons (0–20 ng/mL) were also run on *in-house*-made LFA strips with SBMSO nanophosphor reporters. A volume of 2 μL of DNA sample and 5 μL of anti-FITC antibody conjugated SBMSO nanophosphors (1 mg/mL) mixed with 43 μL of running buffer (50 mM NaCl, 0.1% Tween 20, 10 mM Tris HCl, 0.25% PVP-40, and 1% BSA, pH 8) were added to the sample pad, and the strip was run for 20 min. The LFA strips were then imaged using an iPhone 5S fitted with a 3-D printed attachment that was designed to fit a universal LFA cartridge (MICA-125; DCN Diagnostics), as shown in S1 Fig. The LFA strips were placed into the universal LFA cartridge and when the cartridge is fully inserted into the attachment the result window of the cartridge is aligned with the rear camera of the iPhone 5S and occupies most of the field of view. A proprietary software application called “Luminostics” running on the iPhone 5S was used to control the iPhone’s flash and rear camera. The app turned on the flash for 3 s to excite the nanophosphors. After termination of the excitation and a further 100 ms delay for consistency, the iPhone’s camera captured images of the emission signals at TL and CL. The camera settings used were ISO 2000 and 0.5 s exposure time for all experiments. The camera captures four images and generates the average result. The images were analyzed in a laptop using NIH ImageJ [22] by measuring the signal intensity of TL and CL (the area under the curve) to calculate the TL/CL intensity ratio [16,17,23]. The iPhone reader is described in more detail in our previous publications [17,24].

Finally, to demonstrate the potential applicability of the smartphone-based RPA-LFA for point-of-care diagnosis of cutaneous leishmaniasis, a dilution series of *Leishmania (V*.*) panamensis* DNA (equivalent to 10^−4^–10^3^ parasites/reaction) in nuclease-free water was run in RPA using the protocol described above. The unpurified RPA reaction mixtures were run both on commercial LFA strips with gold nanoparticles and on LFA strips with SBMSO reporters as described above and the limit of detection for gold nanoparticle-based LFA was compared to the LOD for the SBMSO nanophosphor-based LFA.

## Results

### Characterization of RPA amplicons from *Leishmania Viannia panamensis* DNA

**Fig 2:**
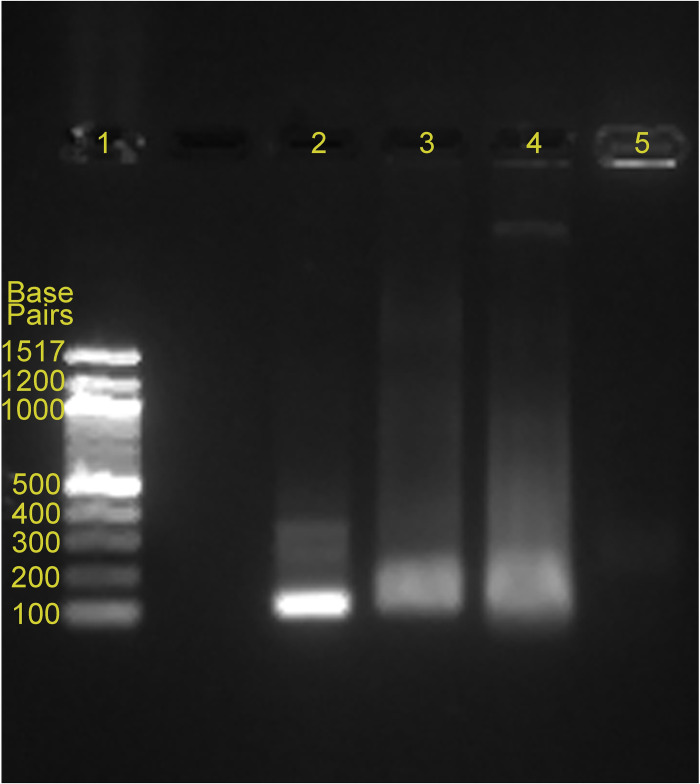
Agarose gel electrophoresis of RPA products: 10 μL of each RPA reaction was loaded on the gel. Lane: (1) DNA ladder (100–1517 base pairs; New England BioLabs Inc.), (2) QIAquick-purified DNA amplicons, (3) Unpurified DNA amplicons sample 1, (4) Unpurified DNA amplicons sample 2, (5) Unpurified negative control RPA reaction mixture with Vero cell DNA.

10 ng of *Leishmania (V*.*) panamensis* DNA (~600 parasites) was used as the template for test samples, and 10 ng Vero cell DNA was used as the template for negative controls in the RPA reactions, using previously-validated DNA primers [5]. Gel electrophoresis of the purified and unpurified RPA reaction mixtures showed DNA amplicons at the expected size (~120 base pairs) and no detectable non-specific products or primer dimers (Fig 2). The unpurified DNA amplicons in the RPA reaction mixture appeared as smears around the expected location on the gel, probably due to the remaining RPA reaction mixture components (Fig 2, lane 3 and lane 4). Lane 5 showed the absence of non-specific amplification with Vero cell DNA (negative control). The purified and unpurified RPA products were quantified using the fluorescent dsDNA binding dye-based QuantiFluor dsDNA system. The concentration of purified *Leishmania* DNA amplicons was estimated at 20.3 ng/μL and that of unpurified *Leishmania* DNA amplicons at 11.7 ng/μL based on a Lambda dsDNA standard curve (S2 Fig).

### Detection of RPA products by lateral flow assay

**Fig 3:**
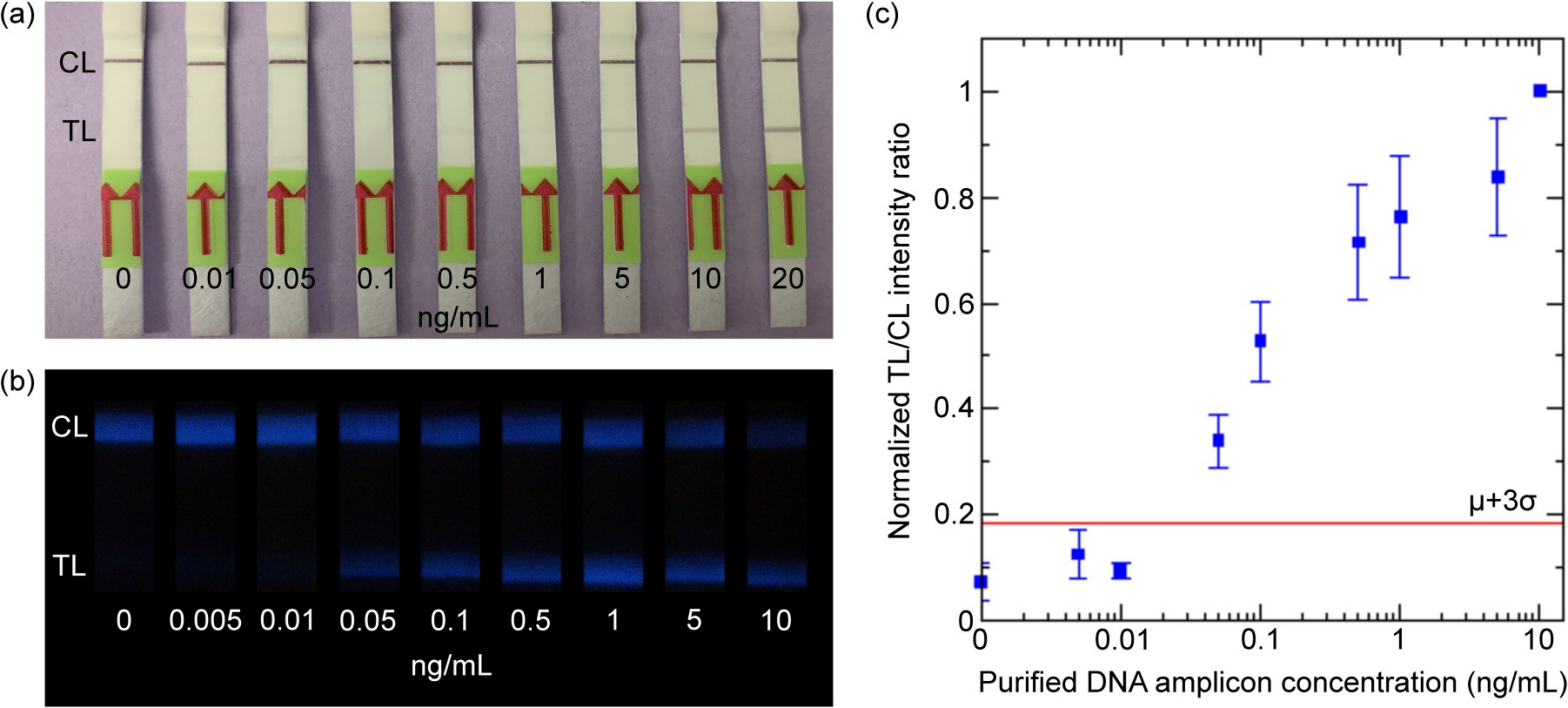
(A) Visual detection of the dilution series of purified RPA products run on commercial gold nanoparticle-based LFA strips. (B) Smartphone images of the dilution series of purified RPA products run on *in-house*-made LFA strips with SBMSO nanophosphor reporters. (C) Normalized TL/CL intensity ratio of SBMSO reporters against the concentration of purified DNA amplicons. Three trials were run for each concentration, then the average was calculated. The red line signifies the detection limit cutoff, taken as the mean plus three times the standard deviation (μ+3σ) of the no-analyte control LFAs.

First, a dilution series of the purified RPA products (0–20 ng/mL) was run with both gold nanoparticle-based LFA and SBMSO nanophosphor-based LFA. The LOD of gold nanoparticle LFA for the purified RPA products was visually estimated to be 5 ng/mL (Fig 3A). The strips with SBMSO nanophosphors were imaged using an iPhone 5S (Fig 3B), and the LOD was estimated as the lowest concentration detected above the cutoff, which is the mean of the no-analyte blank plus three times the standard deviation (μ+3σ) of the blank, as shown in Fig 3C. The LOD of purified RPA products with SBMSO nanophosphors was 0.05 ng/mL, 100-fold lower than that of LOD with gold nanoparticles.

**Fig 4:**
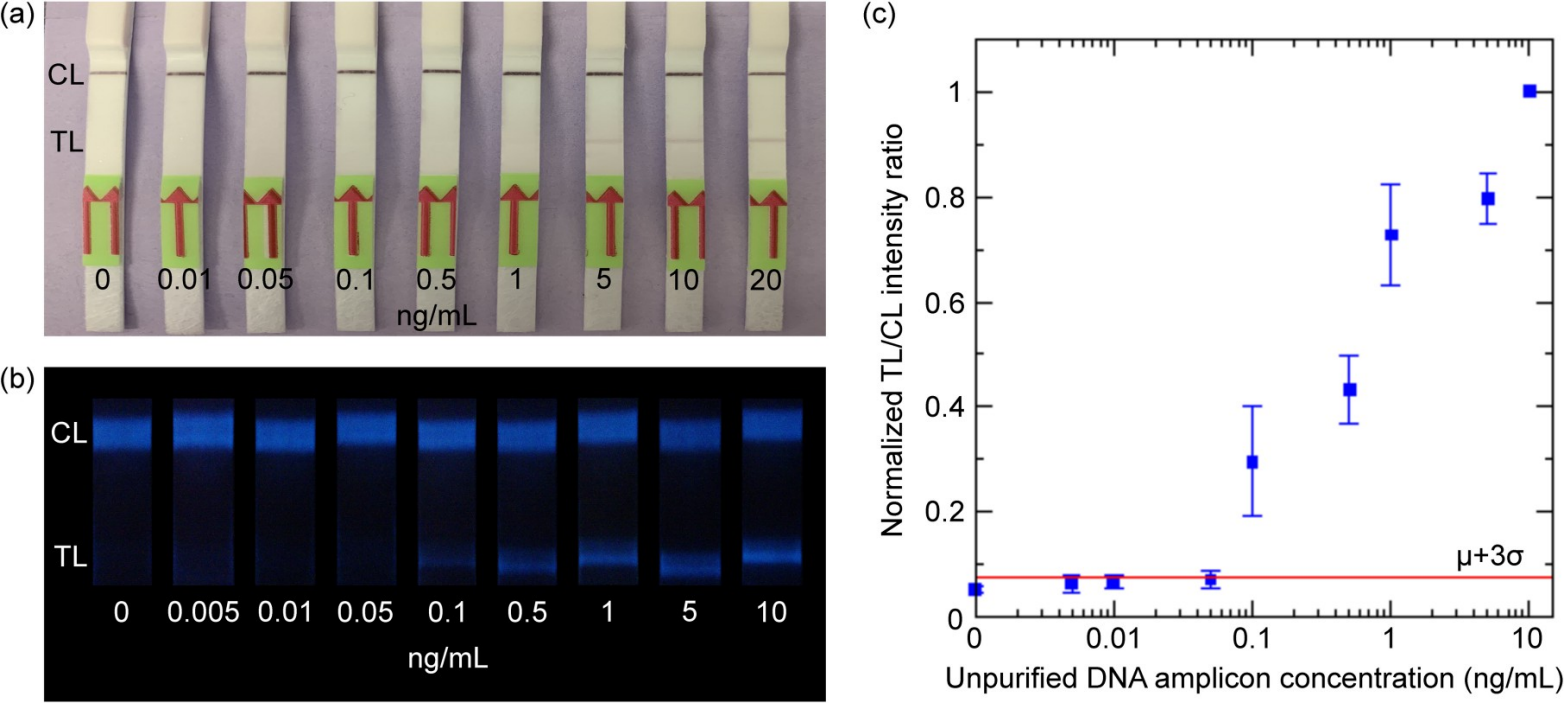
(A) Visual detection of the dilution series of unpurified RPA products run on commercial gold nanoparticle-based LFA strips. (B) Smartphone images of the dilution series of unpurified RPA products run on *in-house*-made LFA strips with SBMSO nanophosphor reporters. (C) Normalized TL/CL intensity ratio of SBMSO reporters against the concentration of unpurified DNA amplicons. Three trials were run for each concentration, then the average was calculated. The red line signifies the detection limit cutoff, taken as the mean plus three times the standard deviation (μ+3σ) of the no-analyte control LFAs.

Next, a dilution series of unpurified RPA products (0–20 ng/mL) was run with both gold nanoparticle and SBMSO nanophosphor LFAs (Fig 4). For the unpurified DNA amplicons, gold nanoparticles showed the same LOD (5 ng/mL) as for the purified amplicons, although the test line bands were dimmer than with the purified product. However, as shown in Fig 4C, with unpurified RPA products, the SBMSO nanophosphors showed an LOD of 0.1 ng/mL, which is two-fold higher than the LOD of purified RPA product (0.05 ng/mL). However, this is still 50-fold lower than the LOD of gold nanoparticle LFA, indicating the higher sensitivity of SBMSO nanophosphors in detecting RPA DNA amplicons.

**Fig 5:**
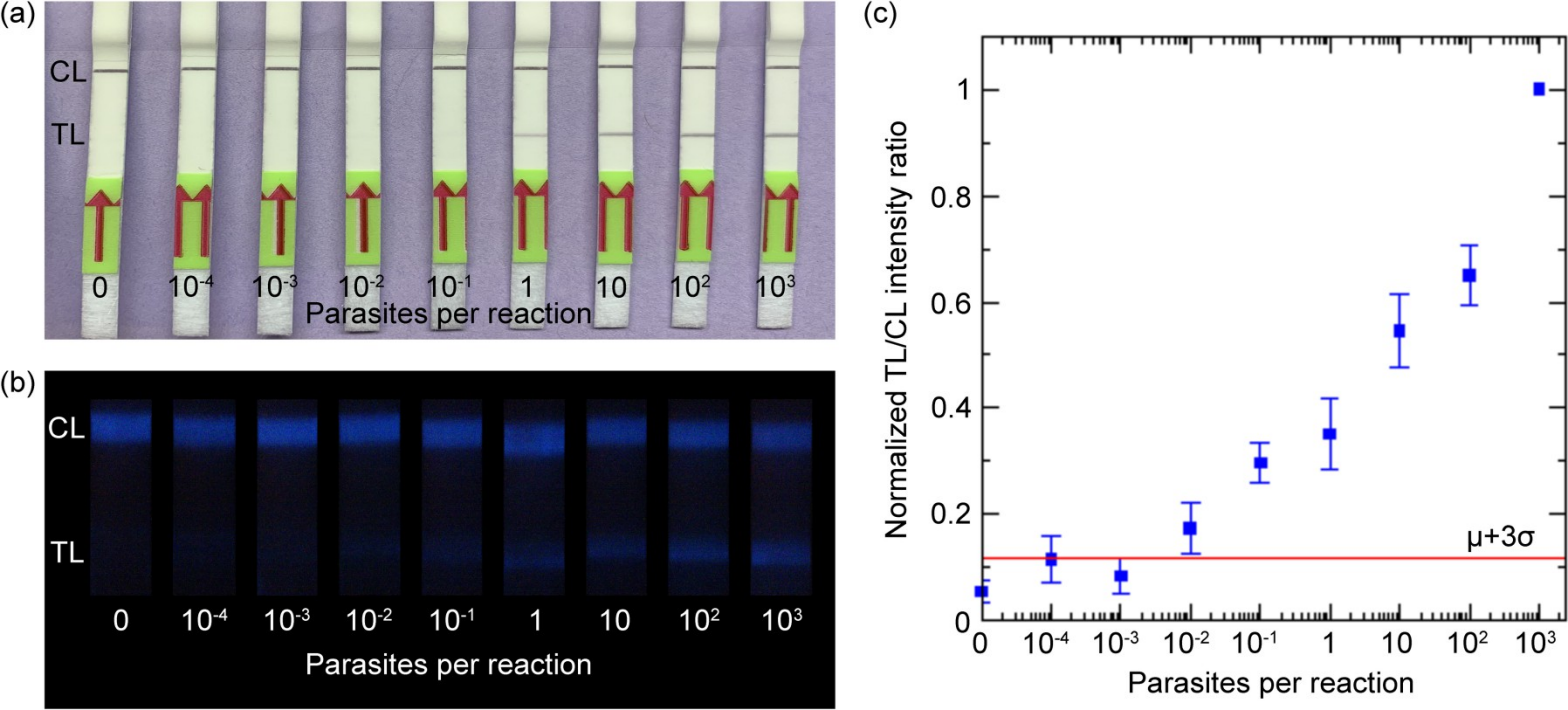
(A) Visual detection of RPA-amplified *Leishmania* parasite DNA dilution series (unpurified RPA products), run on commercial gold nanoparticle-based LFA strips. (B) Smartphone images of the RPA-amplified *Leishmania* parasite DNA dilution series (unpurified RPA products), run on *in-house*-made LFA strips with SBMSO nanophosphor reporters. (C) Normalized TL/CL intensity ratio of SBMSO reporters against the number of parasites added per RPA reaction. Three trials were run for each concentration, and the average was calculated. The red line signifies the detection limit cutoff, taken as the mean plus three times the standard deviation (μ+3σ) of the no-analyte control LFAs.

As the next step, the feasibility of gold nanoparticle-based and nanophosphor-based RPA-LFA for POC diagnosis of cutaneous leishmaniasis was tested by first serially diluting the DNA template and then performing the RPA-LFA. A dilution series of *Leishmania* parasite DNA ranging from 10^−4^ to 10^3^ parasites/reaction were used as the template for RPA reactions and the RPA products were run on gold nanoparticle-based LFA (Fig 5A) and SBMSO nanophosphor-based LFA (Fig 5B). The gold nanoparticles showed an LOD of 1 parasite per reaction, while the SBMSO nanophosphors showed an LOD of 0.01 parasite per reaction (Fig 5C), making SBMSO nanophosphors 100-fold more sensitive.

## Discussion

In this study, we developed an isothermal RPA-LFA workflow using SBMSO nanophosphor LFA reporters for improved diagnostic sensitivity of cutaneous leishmaniasis compared to our previously-developed RPA-LFA with gold nanoparticles. SBMSO nanophosphors are a persistent luminescent material that generates a photon emission lasting several minutes after photoexcitation, compared to the nanosecond lifetimes of most fluorescent materials. The longer lifetime allows time-resolved separation of the emission signal from excitation light by a delayed capture of the luminescence, decreasing background interference and eliminating the need for continuous excitation and expensive optical filters. Nanophosphors also show great photostability [15,16]. We have previously demonstrated the superior performance of persistent luminescent LFA reporters in antigen detection LFAs readable by a smartphone [16,17,24,25]. Here we demonstrate for the first time the use of *in-house* synthesized nanophosphors in the LFA detection of amplified RPA products without requiring post-amplification DNA purification.

The limit of detection of RPA with gold nanoparticle-based LFA readout is estimated at 1 parasite per reaction, but LOD can be 100-fold better, 0.01 parasites per reaction, for LFA based on SBMSO nanophosphors.

This study used RPA primers targeting polyploid kDNA minicircles and amplifying the conserved region of *Leishmania (V*.*) panamensis* [5]. The mitochondrial DNA of protist parasites of the class *Kinetoplastida* exists as a large nucleoid consisting of two circular concatenated genomes; maxicircles (approximately 20–50 copies per parasite) and minicircles (10–20,000 copies per parasite) [26]. *Leishmania* is a member of the *Kinetoplastida* class that contains ~10,000 kDNA minicircles per parasite, each comprising a conserved region of ~120 base pairs [27,28]. Gel electrophoresis of the amplicons shows bands at the expected size (~120 base pairs) and the absence of non-specific amplification, confirming successful RPA amplification. As expected, the unpurified amplicons in the RPA reaction mixture appeared as smears, since proteins and DNA crowding agents in the RPA mixture are known to affect amplicon migration [9,29].

The RPA-LFA shows a high sensitivity compared to conventional microscopic diagnostic methods, and we conducted a preliminary study using 26 Lesion samples (obtained from the Volta region of Ghana) to compare the sensitivity of RPA-LFA and quantitative PCR. Among the 26 samples, 19/26 (73%) had concordant results when comparing the two diagnostic methods [30]. In this study, RPA-LFA results with purified DNA amplicons show that anti-FITC antibody-conjugated SBMSO reporters can successfully bind with amplified *Leishmania* DNA in the RPA product. SBMSO reporters show somewhat less sensitivity in the detection of unpurified DNA amplicons compared to purified DNA amplicons, potentially due to the presence of excess primers, proteins, and RPA reagents in the unpurified RPA products [9]. These results, however, also show that the remaining RPA reaction mixture components do not lead to substantial non-specific binding on LFA. This result is significant because it suggests the possibility to use unpurified products from RPA reactions, avoiding the complexity and cost of purification steps. In addition, this assay uses a minimal volume of the RPA product (2 μL), suggesting the possibility of using RPA reactions with smaller amounts of RPA reagents for each reaction, thereby reducing cost. TwistAmp Basic kit (TwistDx) was used for the RPA reaction and each reaction (50 μL) costs ~USD 5 which is sufficient to run 25 LFA strips. Therefore, the RPA cost per LFA strip is estimated to be ~USD 0.20. Moreover, the LOD of SBMSO reporters is 0.01 parasite per reaction in the detection of RPA-amplified *Leishmania* parasite DNA compared to the 1 parasite per reaction LOD of gold nanoparticles. These results show that the nanophosphor reporters are 100 times more sensitive than commercial gold nanoparticles in detecting *Leishmania* parasite DNA amplicons, suggesting their suitability for more sensitive and cost-effective point-of-care tests for the diagnosis of infectious diseases. The anti-FITC labeled nanophosphors developed here can be used as universal reporters to detect amplicons from any RPA reaction with FITC/FAM labeled primers, and already-existing primer sequences could easily extend nanophosphor-based RPA-LFA to the diagnosis of other protozoan parasite infections, such as visceral leishmaniasis or *Trypanosoma cruzi* infections, which affect humans and animals in the Americas [31]. Therefore, we believe further development of this modular approach can enable more sensitive, fast, and accurate molecular tests for diagnosis of other infectious diseases (e.g., influenza, RSV, SARS-CoV-2) by using nanophosphor reporters, especially in low-resource settings.

This initial proof-of-concept study has some limitations that will be addressed in future studies. The evaluation of sensitivity of the test was based on only one species of *Leishmania*, but multiple species circulate in different endemic areas, which will have to be evaluated using distinct genus or subgenus primer sets. In addition, this study was done using parasite DNA from a single patient sample, and therefore needs further validation using clinical samples of several DNA isolates from different patients and considering non-endemic controls to better estimate the sensitivity and specificity of this assay. Exclusive dependence on the commercial RPA reagent source is a potential liability that could be addressed by evaluating alternative RPA mixtures (when alternative suppliers eventually emerge) or other isothermal amplification methods [32]. Also, DNA extraction needs to be performed at point-of-care without requiring DNA purification prior to amplification (e.g., using heat to lyse the cells and release the nucleic acids). Recent technological advances could facilitate the integration of the isothermal amplification and LFA into a single, disposable, and low cost cartridge within a small enclosed microfluidic device to make this technique appropriate for the use in POC settings [33,34]. It also is noteworthy that smartphone-based reading of RPA-LFA results allows fast transmission of the results from field locations to healthcare providers, enabling prompt medical decisions and efficient treatment.

## Supporting information

S1 Fig A 3-D printed phone accessory with minimal optical hardware, containing a lens and a bundle of inexpensive plastic optical fibers but no electronic components, was used as a dark imaging compartment which was designed to hold a universal LFA cartridge (MICA-125; DCN Diagnostics) such that the result window of the cartridge is aligned with the rear camera of the iPhone 5S and occupies most of the field of view when the cartridge is fully inserted into the attachment. A proprietary software application, “Luminostics”, controls the flash and the rear camera of the iPhone. The flash excites the nanophosphors for ~3 s, and, after switching off the flash, the camera captures the images after a ~100 ms time delay. The camera captures four images and generates the average result. We have described the iPhone reader in more detail in our previous publications [17,24].
(TIF)

S2 Fig dsDNA standard curve obtained from the QuantiFluor dsDNA system. The inset shows the fluorescence obtained with 4 μL of 5X diluted purified and unpurified RPA products (in red) and their respective dsDNA concentrations. According to the standard curve, the dsDNA amount of purified and unpurified samples is 16.23 and 9.37 ng/well, respectively. Therefore, the dsDNA concentration of the undiluted purified and unpurified amplicons is 20.3 and 11.7 ng/μL, respectively.
(TIF)
