# Supplementary figures and images for: Smartphone-readable RPA-LFA for the high-sensitivity detection of Leishmania kDNA using nanophosphor reporters
Source: PLoS Negl Trop Dis. 2023 Jul 3;17(7):e0011436. doi: 10.1371/journal.pntd.0011436 (PMC10353800; doi:10.1371/journal.pntd.0011436)

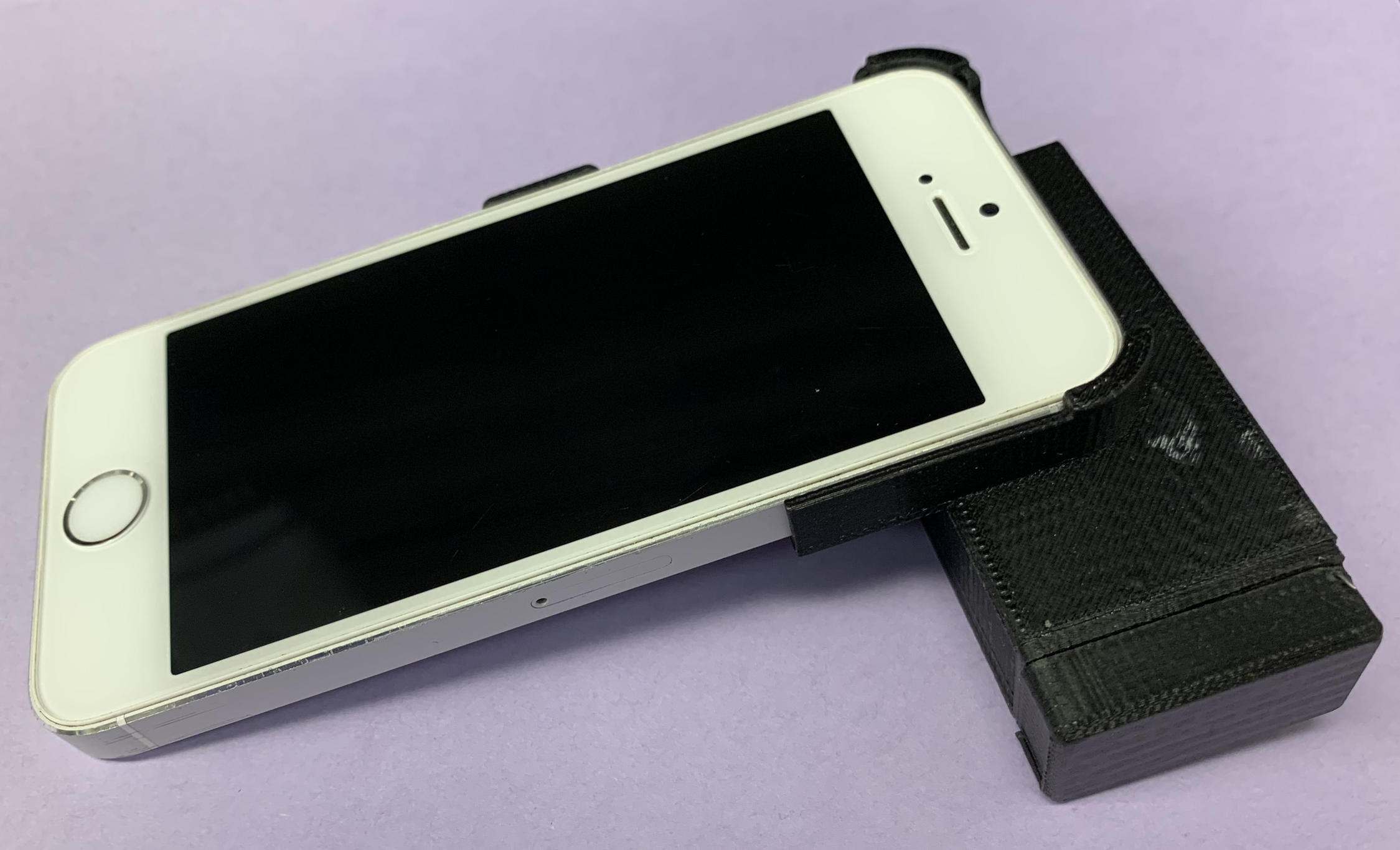

Supplement: S1 Fig — A proprietary software application, “Luminostics”, controls the flash and the rear camera of the iPhone. The flash excites the nanophosphors for ~3 s, and, after switching off the flash, the camera captures the images after a ~100 ms time delay. The camera captures four images and generates the average result. We have described the iPhone reader in more detail in our previous publications [17,24]. (TIF) [file pntd.0011436.s001.tif]

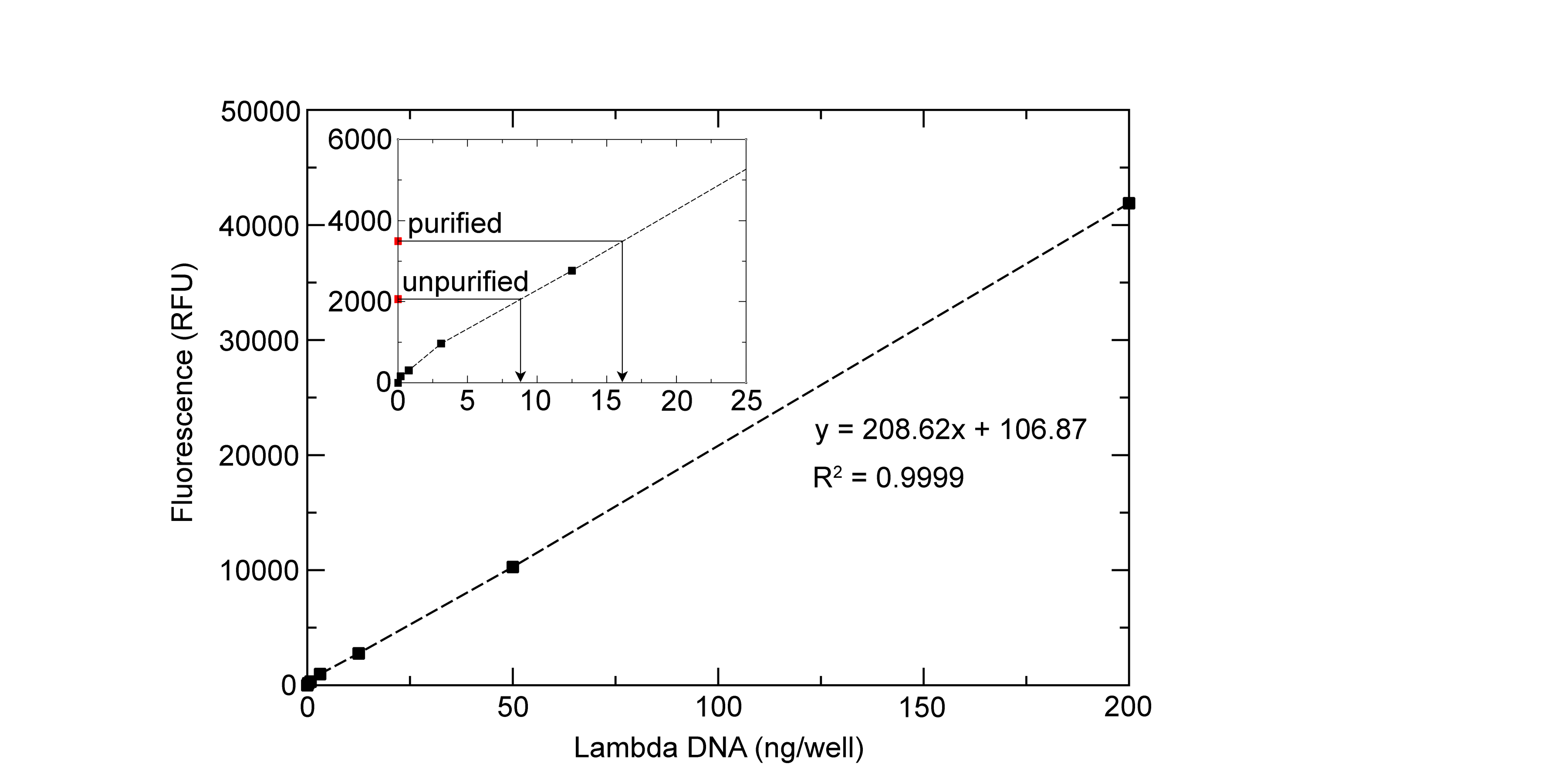

Supplement: S2 Fig — The inset shows the fluorescence obtained with 4 μL of 5X diluted purified and unpurified RPA products (in red) and their respective dsDNA concentrations. According to the standard curve, the dsDNA amount of purified and unpurified samples is 16.23 and 9.37 ng/well, respectively. Therefore, the dsDNA concentration of the undiluted purified and unpurified amplicons is 20.3 and 11.7 ng/μL, respectively. (TIF) [file pntd.0011436.s002.tif]
